# Benzoic acid inhibits Coenzyme Q biosynthesis in *Schizosaccharomyces pombe*

**DOI:** 10.1371/journal.pone.0242616

**Published:** 2020-11-24

**Authors:** Ikuhisa Nishida, Ryota Yanai, Yasuhiro Matsuo, Tomohiro Kaino, Makoto Kawamukai

**Affiliations:** 1 Department of Life Sciences, Faculty of Life and Environmental Sciences, Shimane University, Matsue, Japan; 2 Institute of Agricultural and Life Sciences, Academic Assembly, Shimane University, Matsue, Japan; Tokyo Daigaku, JAPAN

## Abstract

Coenzyme Q (CoQ, ubiquinone) is an essential component of the electron transport system in aerobic organisms. Human type CoQ_10_, which has 10 units of isoprene in its quinone structure, is especially valuable as a food supplement. Therefore, studying the biosynthesis of CoQ_10_ is important not only for increasing metabolic knowledge, but also for improving biotechnological production. Herein, we show that *Schizosaccharomyces pombe* utilizes *p*-aminobenzoate (PABA) in addition to *p*-hydroxybenzoate (PHB) as a precursor for CoQ_10_ synthesis. We explored compounds that affect the synthesis of CoQ_10_ and found benzoic acid (Bz) at >5 μg/mL inhibited CoQ biosynthesis without accumulation of apparent CoQ intermediates. This inhibition was counteracted by incubation with a 10-fold lower amount of PABA or PHB. Overexpression of PHB-polyprenyl transferase encoded by *ppt1* (*coq2*) also overcame the inhibition of CoQ biosynthesis by Bz. Inhibition by Bz was efficient in *S*. *pombe* and *Schizosaccharomyces japonicus*, but less so in *Saccharomyces cerevisiae*, *Aureobasidium pullulans*, and *Escherichia coli*. Bz also inhibited a *S*. *pombe ppt1* (*coq2*) deletion strain expressing human *COQ2*, and this strain also utilized PABA as a precursor of CoQ_10_. Thus, Bz is likely to inhibit prenylation reactions involving PHB or PABA catalyzed by Coq2.

## Introduction

Coenzyme Q (CoQ), also called ubiquinone, is a component of the electron transport chain that participates in aerobic respiration in eukaryotes and most prokaryotes [1]. CoQ consists of a quinone ring and a hydrophobic isoprenoid side chain that has an all-trans configuration and a certain number of isoprene units [2]. The quinone moiety is reduced to form CoQH_2_ (ubiquinol) from CoQ (ubiquinone), an essential component of electron transfer and oxidation-reduction enzymes, and an important antioxidant [3]. A CoQ-producing organism produces one type of CoQ as a main product, which is classified according to the length of the isoprenoid side chain [4]. For example, *Homo sapiens* and *Schizosaccharomyces pombe* predominantly produce CoQ_10_ with 10 isoprene units, whereas *Mus musculus* and *Arabidopsis thaliana* produce CoQ_9_, *Escherichia coli* produces CoQ_8_, and *Saccharomyces cerevisiae* produces CoQ_6_ [5]. The side chain length of CoQ is determined by species-specific polyprenyl diphosphate synthases [6, 7], which utilize as substrates isopentenyl pyrophosphate and farnesyl pyrophosphate derived from the mevalonate (MVA) pathway in eukaryotes or archaea and the methylerythritol phosphate (MEP) pathway in bacteria and several photosynthetic eukaryotes [2]. The main precursor of the benzoquinone ring is *p*-hydroxybenzoate (PHB), which is derived from chorismic acid in prokaryotes and tyrosine in eukaryotes [8]. The biosynthetic pathway for the complete conversion of PHB to CoQ in eukaryotes consists of at least eight steps (S1 Fig). After polyprenyl diphosphate is synthesized, it is transferred to PHB by Coq2 (PHB-polyprenyl diphosphate transferase; Ppt1). The six-membered ring of prenylated PHB is then modified by three hydroxylations catalyzed by Coq6, Coq7, and a still-unidentified enzyme(s), two *O*-methylations catalyzed by Coq3, *C*-methylation catalyzed by Coq5, and decarboxylation catalyzed by an unknown enzyme(s) [8]. In eukaryotes, this pathway has been most comprehensively studied in *S*. *cerevisiae* [9], *S*. *pombe* [10], and various animals [11, 12]. At least 10 genes (*COQ1*–*COQ9* and *COQ11*) in *S*. *cerevisiae* [13] and 11 genes (*dps1*, *dlp1*, *ppt1*, *coq3–coq9*, and *coq11*) in *S*. *pombe* are required for CoQ biosynthesis [2, 14–16]. Importantly, except for *coq11*, homologous genes are present in human [10, 13]. However, the functions of *COQ4*, *COQ8*, *COQ9*, and *COQ11* have not yet been clearly resolved [17, 18], and the pathway upstream of PHB synthesis is only partially understood [19].

Studies using *S*. *cerevisiae* have revealed that stable isotope-labeled *p*-aminobenzoate (PABA) and PHB are incorporated into the quinone ring of CoQ_6_ [20, 21]. However, it is not clear how widely PABA is utilized as a precursor for CoQ synthesis in other species.

Exploring inhibitors or inducers of CoQ biosynthesis strengthens our understanding of CoQ metabolism, and paves the way for modulating the cellular level of CoQ using drugs. Some studies have reported inhibitors of CoQ biosynthesis. For example, 4-nitrobenzoic acid (4-NB) is an efficient inhibitor of CoQ biosynthesis that acts by inhibiting PHB-polyprenyl transferase (COQ2) in mammalian cells [22, 23], chlorobenzoic acid is also thought to inhibit the same reaction [24, 25], and inhibitors of COQ7 have also been identified [26]. Vanillic acid was reported to bypass the requirement for the reaction involving COQ6 [27], while resveratrol was found to induce genes involved in CoQ biosynthesis without increasing CoQ synthesis in rats [28]. Thus, our knowledge of inhibitors and inducers of CoQ biosynthesis remains limited.

In the present study, we showed that PABA is utilized as a precursor for quinone ring formation in *S*. *pombe*, investigated inhibitors of CoQ synthesis, and demonstrated that benzoic acid (Bz) is a specific inhibitor of CoQ biosynthesis in *S*. *pombe*

## Materials and methods

### Fungi strains, *E*. *coli* strains, and culture media

Fungi and *E*. *coli* strains used in this study are listed in Table 1. Standard yeast culture media and genetic methods were as described previously [29]. *S*. *pombe* strains were grown in complete YES medium comprising 0.5% OXOID yeast extract (Hampshire, UK) (w/v), 3% glucose (w/v), and 225 mg/L each of adenine sulfate, leucine, uracil, histidine, and lysine hydrochloride. OXOID yeast extract lot number 2198213–02 was employed in all experiments because *S*. *pombe* cell density was five times higher (~10^8^) with this lot than with other lots (LOT 2665431–02 and LOT 1448470–04). Non-fermentable carbon source medium (YEGES) was prepared by adding 2% glycerol (w/v) and 1% ethanol (w/v) instead of 3% glucose (w/v) to YES medium. For synthetic medium, Pombe Minimal medium (PM) with 75 mg/L uracil was used as necessary. The pREP41 vector containing the relatively weak promoter (*nmt41*) of the thiamine-repressible gene *nmt1* of *S*. *pombe* [30] was used to overexpress the *ppt1* gene. Wild-type (WT) cells transformed with pREP41 or pREP41-PPT1OR [31] were selected on PMU (PM containing uracil but lacking leucine) containing 10 μM thiamine and streaked onto the same media. For moderate *ppt1* overexpression, transformants on the plate were grown in PMU liquid media containing 0.15 μM thiamine for 1 day at 30°C. Cells were washed three times and transferred into PMU with or without 0.15 μM thiamine and incubated for 2 days at 30°C. *S*. *cerevisiae* and *A*. *pullulans* cells were grown in complete YPD medium comprising 1% yeast extract (w/v), 2% glucose (w/v), and 2% HIPOLYPEPTON S (w/v). *E*. *coli* cells were grown in complete LB medium comprising 0.5% yeast extract (w/v), 1% NaCl (w/v), and 1% HIPOLYPEPTON S (w/v).

**Table 1:** Microorganisms used in this study.

Strain	Genotype	Source
*S*. *pombe* L972	*h*^-^	Lab stock
*S*. *pombe* PR110	*h*^+^ *ura4*-*D18 leu1*-*32*	P. Russell
*S*. *pombe* KH2 (OG1)	*h*^+^ *ura4*-*D18 leu1*-*32 ppt1*::*kanMX6*	[10]
*S*. *pombe* KH4 (LV974)	*h*^+^ *ura4*-*D18 leu1*-*32 coq4*::*kanMX6*	[10]
*S*. *japonicus* NIG2021	*h*^90^	National Institute of Genetics
*S*. *japonicus* Kinzaki in Matsue City	*h*^90^	[38]
*S*. *cerevisiae* BY4741	*MAT*a *leu2*Δ0 *ura3*Δ0 *his3*Δ1 *met15*Δ0	Lab stock
*A*. *pullulans* EXF-150	Homothallic	University of Ljubljana [43]
*E*. *coli* DH5α	F^-^ Φ80d*lacZ*ΔM15 Δ(*lacZYA-argF*)U169 *deoR recA*1 *endA*1 *hsdR*17(r_K_^-^, m_K_^+^) *phoA supE*44 λ^-^ *thi*-1 *gyrA*96 *relA*1	Lab stock

### Sources of aromatic compounds

Chemicals were obtained from the following companies: 4-aminobenzoic acid and 4-hydroxybenzoic acid were from Wako Pure Chemical Industries, Ltd. (Tokyo, Japan) (015–02332 and 088–04105, respectively); 4-aminobenzoic acid (ring-^13^C_6_, 99%) and 4-hydroxybenzoic acid (ring-^13^C_6_, 99%) were from Cambridge Isotope Laboratories, Inc. (Cambridge, UK) (CLM-1541-PK and CLM-4745-PK, respectively); benzoic acid and sodium benzoate were from NACALAI TESQUE INC. (Kyoto, Japan) (04120–52 and 31211–22, respectively); 4-chlorobenzoic acid, 2,4-dihydroxybenzoic acid, and 4-nitrobenzoic acid were from Tokyo Chemical Industry Co., Ltd. (Tokyo, Japan) (C0134, D0568, and N0156, respectively).

### CoQ extraction and measurement

Fungi cells were pre-cultured in 10 mL of the indicated liquid medium for 1 day at 30°C. *E*. *coli* cells were pre-cultured in 10 mL of LB for half a day at 37°C. Each pre-culture was inoculated into a larger volume of medium, and the main culture was grown for the indicated time. Cell counts was measured using a Sysmex CDA-1000B cell counter (Sysmex, Tokyo, Japan) and optical density (OD) values were measured using a Shimadzu UVmini-1240 spectrophotometer (Shimadzu, Kyoto, Japan). At the indicated times, cells were harvested, and CoQ was extracted as described previously [10]. The CoQ crude extract was analyzed by normal-phase thin-layer chromatography (TLC) with authentic CoQ_6_ or CoQ_10_ standards. Normal-phase TLC was conducted on a Kieselgel 60 F_254_ plate (Merck Millipore, MA, USA) and developed with benzene. The plate was viewed under UV illumination, the CoQ band was collected, and samples were extracted with hexane/isopropanol (1:1, v/v). Samples were then dried and solubilized in ethanol. Purified CoQ was subjected to high-performance liquid chromatography on a Shimadzu HPLC Class *VP* series instrument (Shimadzu) equipped with a reversed-phase YMC-Pack ODS-A column (A-312-3 AA12S03-1506PT, 150 × 6 mm, internal diameter 3 μm,120A, YMC, Kyoto, Japan). Ethanol was used as the mobile phase at a flow rate of 1.0 mL/min, and detection of CoQ was performed by monitoring absorption at 275 nm.

### Measurement of CoQ by liquid chromatography-mass spectrometry (LC-MS)

*S*. *pombe* cells cultured in YES medium for 1 day were transferred to fresh YES medium and cultured at 30°C for 2 days. The initial cell density in YES was 1×10^5^ cells/mL. CoQ was extracted as described above. LC-MS analysis was performed using a Xevo-TQ mass spectrometer (Waters, MA, USA) coupled to an ESCi multi-mode ionization source (Waters) that combines electrospray ionization (ESI) and atmospheric pressure chemical ionization (APCI). CoQ and related compounds were analyzed by APCI in positive mode (APCI+). Data acquisition and processing were performed using a MassLynx system (Waters). To detect the fragmented quinone ring of CoQ, LC-MS/MS was carried out using the product-ion-scan mode, and *m*/*z* 881, 887, and 867 ions of [M+NH_4_]^+^ forms were selected as precursor ions for CoQ_10_, ring-^13^C_6_-CoQ_10_, and putative 2-methoxy-4-hydroxy-5-decaprenyl-benzoic acid, respectively. The conditions are listed in S1 Table.

### Antibodies

To immunochemically detect CoQ biosynthetic proteins, rabbit polyclonal antisera were prepared by Sigma-Aldrich by injecting rabbits with specific peptides of Coq proteins [32]. The specificity of antisera against each of the CoQ biosynthetic proteins (Dlp1, diluted 1:1000; Coq4, diluted 1:500; Coq8, diluted 1:1000) was assessed by western blot analysis. Preparation of cell lysates and detection of CoQ biosynthetic proteins by immunoblotting *S*. *pombe* cell lysates were performed as described previously [33]. WT *S*. *pombe* (PR110) cells were inoculated into 55 mL YES main cultures with or without Bz (initial cell density ~1×10^5^ cells/mL) and incubated with rotation at 30°C for 2 days, and then harvested. For mitochondria isolation, WT *S*. *pombe* (PR110) cells were cultivated in 1.5 L YES or YES with 25 μg/mL Bz (initial OD_600_ ~0.05, cultivated for 20 h with rotation at 30°C) and mitochondria-enriched samples were prepared as described previously [34]. Lysate proteins were separated by SDS-PAGE, after which western blot analysis was performed using an ECL detection system (GE Healthcare, IL, USA). Rabbit polyclonal antibodies against the PSTAIRE peptide (Cdc2, diluted 1:1000) were purchased from Santa Cruz Biotechnology. Horseradish peroxidase-conjugated anti-rabbit IgG antibody (Promega, WI, USA) was used as secondary antibody (diluted 1:2000). These antibodies were dissolved in a Can Get Signal immunoreaction enhancer solution (TOYOBO, Osaka, Japan). For quantification of protein bands, Image J (https://imagej.nih.gov/ij/download.html) was used.

### Data and statistical analyses

All experiments were performed at least three times, and average values and standard deviation (SD) were calculated except for S7 and S8 Figs. Data from control and target samples were compared using the two-sample *t*-test in Microsoft Excel (Microsoft, WA, USA), and *p*-values <0.05 were considered statistically significant.

## Results

### *S*. *pombe* utilizes PABA as a precursor in CoQ synthesis

**Fig 1:**
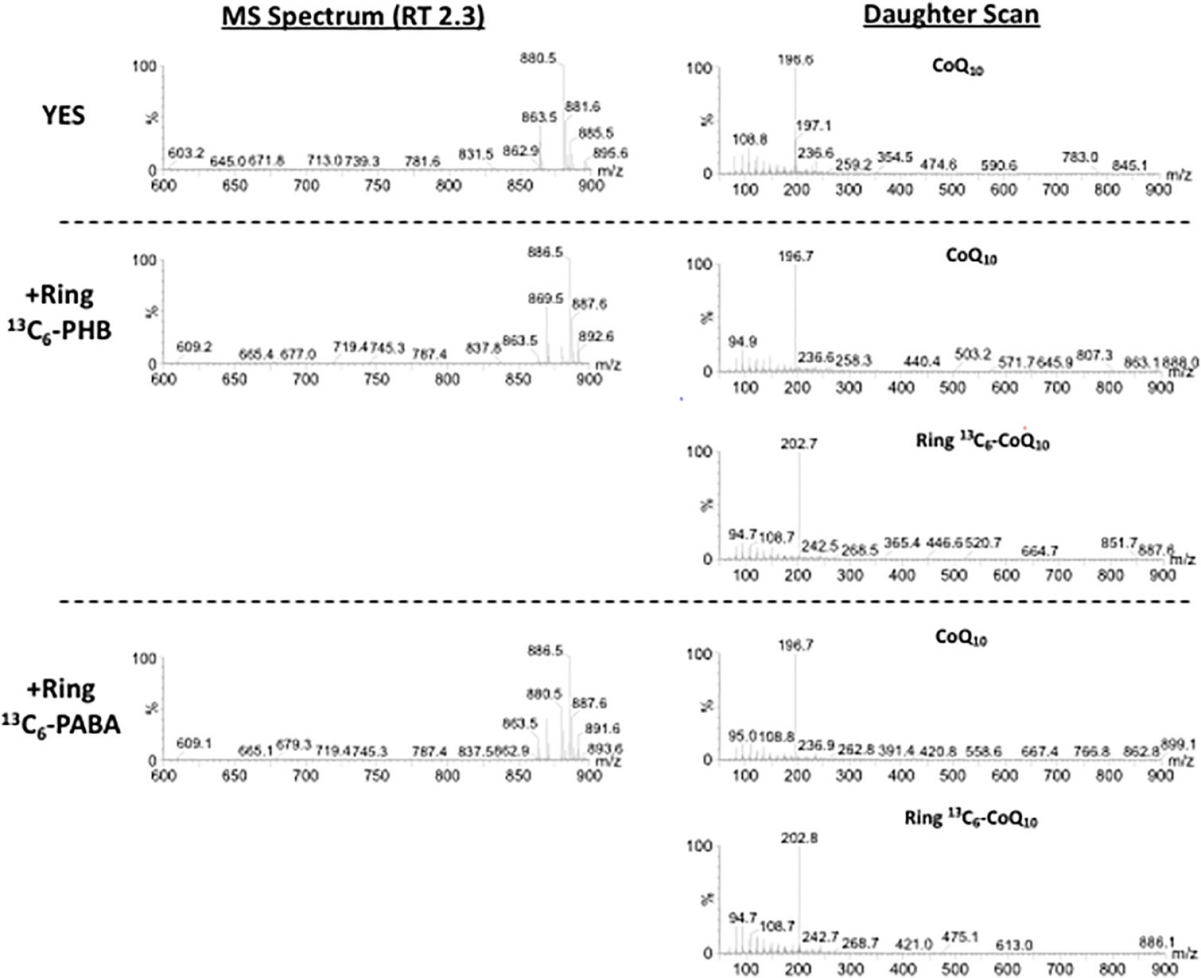
PABA and PHB are metabolized to supply quinone for CoQ_10_ synthesis in *S*. *pombe*. *S*. *pombe* wild-type (WT) PR110 cells were pre-cultivated in 10 mL YES medium for 1 day, 1 μg/mL of ^13^C_6_-PABA or ^13^C_6_-PHB was added to 55 mL of YES media containing 1×10^5^ cells/mL, and the cells were cultivated for 2 days with rotation at 30°C. CoQ_10_-enriched samples were obtained after separation of lipids by TLC, and samples were subjected to LC-MS and LC-MS/MS (daughter scan) analyses to detect stable isotope-labeled CoQ_10_.

In addition to PHB, PABA is utilized as a precursor in CoQ synthesis in *S*. *cerevisiae* [20, 21, 35], the sole species known to utilize PABA for CoQ synthesis. Therefore, we first tested whether PABA is also utilized in *S*. *pombe*. ^13^C_6_ labeled-PABA or ^13^C_6_ labeled-PHB was incubated with the *S*. *pombe* PR110 strain and the lipid fraction was extracted. After the CoQ_10_-enriched fraction was separated by TLC, ^13^C_6_-CoQ_10_ was measured by LC-MS. When 1 μg/mL ^13^C_6_-PHB was incubated, ^13^C_6_-labeled CoQ_10_, which yields an [M+NH_4_]^+^ ion product with a mass 6 Da (886.5) higher than that of none-labeled CoQ_10_ [M+NH_4_]^+^ (880.5), was detected by MS (Fig 1). After fragmentation of this product, a tropylium ion derivative, an aromatic species with the formula [C_7_H_7_]^+^, was generated. As a result, an [M]^+^ ion with a mass of 202.7, which has a mass 6 Da higher than that of the non-labeled tropylium ion [M]^+^ (196.7), was detected. About 88% of the total CoQ pool was labeled with ^13^C_6_ derived from ^13^C_6_-PHB. Similarly, when cells were incubated with ^13^C_6_-PABA, a ^13^C_6_-CoQ_10_ product with a 6 Da increase was detected. About 60% of the total CoQ pool was labeled with ^13^C_6_ derived from ^13^C_6_-PABA. This result shows that PABA was efficiently utilized as a precursor of CoQ synthesis in *S*. *pombe*, similar to *S*. *cerevisiae*. Additionally, exogenous ^13^C_6_-PHB was more efficiently incorporated in CoQ_10_ than ^13^C_6_-PABA.

### Bz is an inhibitor of CoQ biosynthesis in *S*. *pombe*

**Fig 2:**
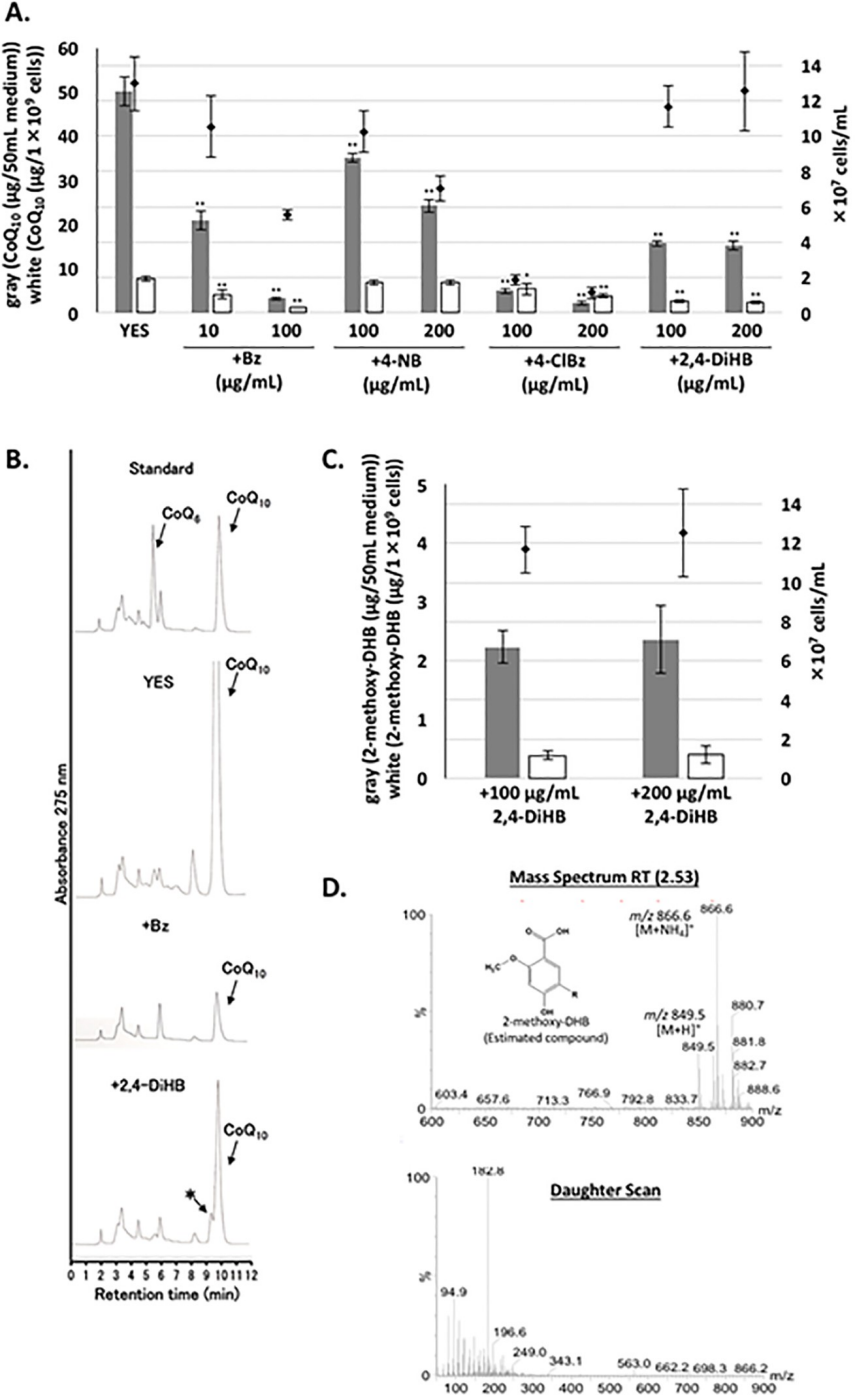
Effect of PABA/PHB analogs on CoQ production. (A) For the pre-culture, WT PR110 cells were cultivated in 10 mL medium for 1 day. The indicated amount (μg/mL) of benzoic acid (Bz), 4-nitrobenzoic acid (4-NB), 4-chlorobenzoic acid (4-ClBz), or 2,4-dihydroxybenzoic acid (2,4-DiHB, β-resorcylic acid) was added to the media containing ~1×10^5^ cells/mL and the cells were cultivated for two days with rotation at 30°C. Gray bars show the CoQ_10_ content per 50 mL of medium, and white bars show CoQ_10_ normalized against cell number. Diamonds show cell number. Five micrograms of CoQ_6_ was used as an internal standard. Data are represented as the mean ± standard deviation (SD) of three measurements. Asterisks on bars denote statistically significant differences (**p<0.01) relative to the amount of CoQ in the medium or cells grown in YES (Student’s *t-*test). (B) CoQ_10_ intermediate-like peak detected by HPLC analysis. (C) Quantitative analysis of the CoQ_10_ intermediate-like peak (*) which is predicted to be 2-methoxy-4-hydroxy-5-decaprenyl-benzoic acid (2-methoxy-DHB). HPLC analysis was performed at 269 nm. (D) For LC-MS/MS analysis, the *m*/*z* 867 ion associated with [M+NH_4_]^+^ selected as the precursor ion for a compound predicted to be 2-methoxy-DHB is shown (-R indicates the decaprenyl moiety).

**Fig 3:**
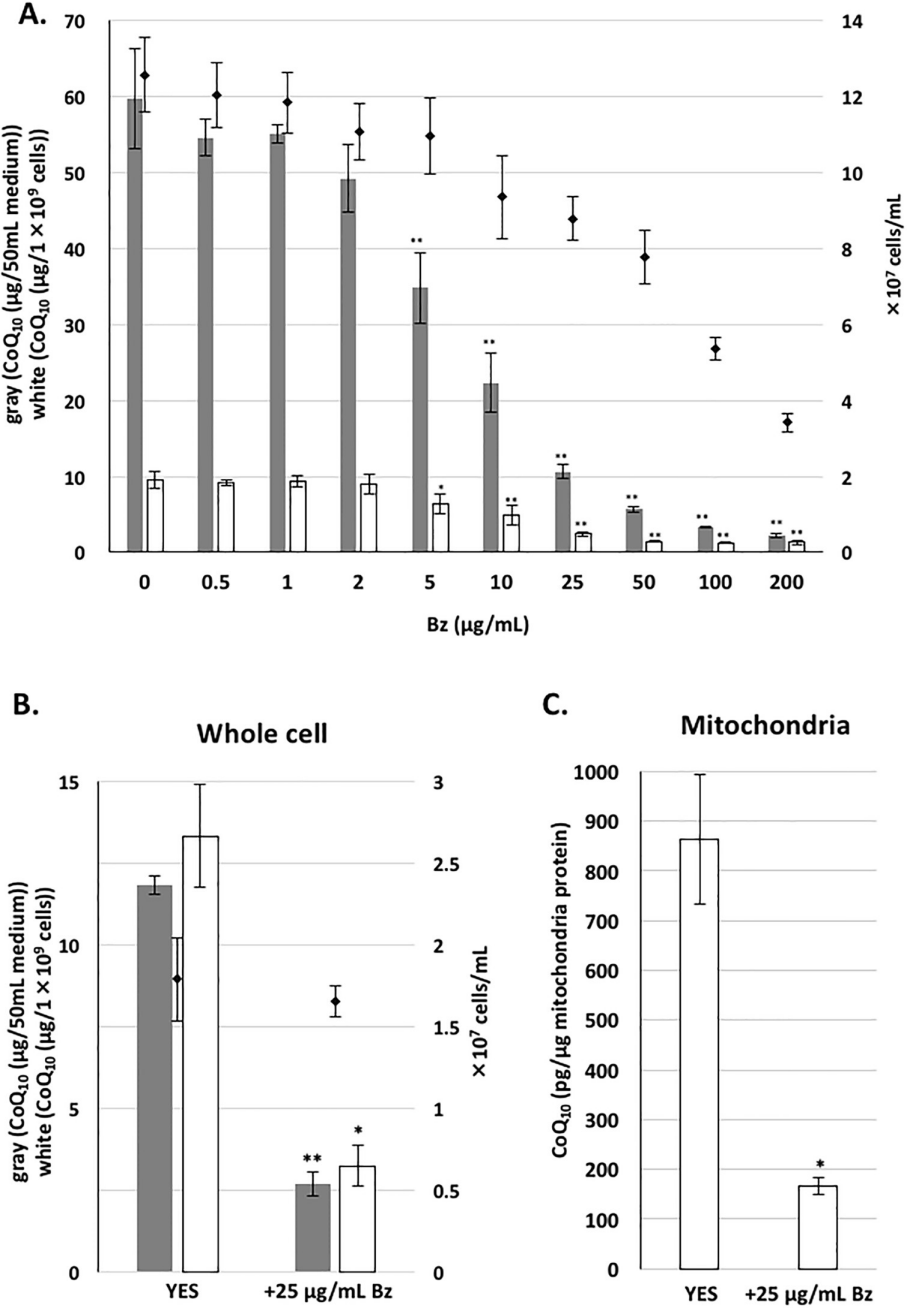
CoQ_10_ production following addition of various concentration of Bz. WT PR110 cells were pre-cultivated in 10 mL YES medium for 1 day. Cells at ~1×10^5^ cells/mL in YES media were cultivated for two days with rotation at 30°C in the presence of the indicated amount (μg/mL) of Bz, or without Bz. Gray bars show the CoQ_10_ content per 50 mL of medium, and white bars show CoQ_10_ normalized against cell number. Diamonds show cell number. Five micrograms of CoQ_6_ was used as an internal standard. Data are represented as the mean ± SD of three measurements. (B) WT PR110 cells were pre-cultivated in 55 mL medium for 1 day. Yeast cells at an initial cell density of OD_600_ 0.05 were cultivated in 1.5 L YES with 25 μg/mL of Bz, or without Bz, for 20 h with rotation at 30°C. Gray bars show the CoQ_10_ content per 50 mL of medium, and white bars show CoQ_10_ normalized against cell number. Diamonds show cell number. Five micrograms of CoQ_6_ was used as an internal standard. Data are represented as the mean ± SD of two measurements. (C) From isolated mitochondria, lipids were extracted with hexane:methanol:isopropanol (5:2:1) and the amount of CoQ was measured by HPLC. Two micrograms of CoQ_6_ was used as an internal standard for CoQ extraction. Protein concentration was measured by a Bio-Rad protein assay kit. (A−C) Asterisks on bars denote statistically significant differences (**p<0.01, *p<0.05) relative to samples from YES without Bz (Student’s *t*-test).

*S*. *pombe* is an excellent microorganism for increasing the production of CoQ_10_ [31, 32], as well as for studying the pathway of CoQ_10_ synthesis, which could lead to the identification of human orthologous enzymes [36, 37]. To obtain a better understating of CoQ_10_ synthesis, we examined analogous compounds of PABA or PHB that may alter CoQ synthesis in *S*. *pombe*. We tested the effect of Bz, 4-nitrobenzoic acid (4-NB), 4-chlorobenzoic acid (4-ClBz), and 2,4-dihydroxy benzoic acid, also known as 2,4-DiHB or β-resorcylic acid (β-RA) (S2 Fig). Although we did not identify a compound that enhanced CoQ_10_ production in *S*. *pombe*, we found that Bz, 4-ClBz, and 2,4-DiHB inhibited CoQ synthesis (Fig 2A). In the case of 2,4-DiHB treatment, an intermediate-like compound, probably 2-methoxy-4-hydroxy-5-decaprenyl-benzoic acid, was accumulated (Fig 2B, 2C and 2D). However, 4-nitrobenzoic acid (4-NB), an inhibitor of COQ2 in mammalian cells [22], did not inhibit CoQ production in *S*. *pombe*, although it moderately inhibited cell growth. Bz treatment most effectively lowered *S*. *pombe* CoQ_10_ production. Bz at 5 μg/mL or higher concentrations significantly decreased the CoQ_10_ level (Fig 3A). Incubation with 10 μg/mL (81.9 μM) Bz and 100 μg/mL (819 μM) Bz resulted in decreases of ~50% and 87% in the CoQ_10_ level (μg/10^9^ cells), respectively. Incubation with 10 μg/mL Bz for 2 days decreased cell number to 74% of that of the controls, but did not affect dry cell weight (DCW) (Table 2). However, incubating with 100 μg/mL Bz for 2 days decreased both cell number and DCW. Significantly, 10 μg/mL Bz and 100 μg/mL Bz decreased CoQ_10_/DCW by 42% and 9%, respectively, compared with cells not treated with Bz. The L972 strain, a WT strain with no auxotrophy (S3 Fig), showed a similar reduction in CoQ_10_ after treatment with 100 μg/mL Bz, indicating that the effect of Bz was not strain-dependent. We did not observe any accumulation of any intermediate compound such as prenylated benzoic acid by MS analysis in the wild type cells incubated with benzoic acid. We tested the effect of Bz on Colony Forming Unit (CFU) of PR110 strain. Bz did not significantly affect CFU (S4A Fig; gray bars), while CoQ_10_ was clearly reduced (S4B Fig), indicating reduction of CoQ_10_ is not due to loss of cell’s viability.

**Table 2:** Amount of CoQ (dry cell weight basis).

Condition	CoQ_10_ (μg)	CoQ_10_ (μg)/10^9^ cells	CoQ_10_ (mg)/g-DCW	mg-DCW
YES	50.1 ± 3.3	7.75 ± 0.43	0.303 ± 0.025	165.5 ± 2.9
+10 μg/mL Bz	20.9 ± 2.0	4.09 ± 1.02	0.126 ± 0.012	165.7 ± 1.3
+100 μg/mL Bz	3.27 ± 0.3	1.18 ± 0.04	0.027 ± 0.002	119.3 ± 6.3

We also measured the amount of mitochondrial CoQ_10_ after separating the mitochondria-enriched fraction by several centrifugation steps, as described in the Materials and Methods. Bz treatment decreased the mitochondrial CoQ_10_ concentration to that equivalent to the decrease in the total cellular CoQ_10_ level (Fig 3B and 3C). Additionally, in order to explore whether Bz promotes the degradation of CoQ_10_, we evaluated the effect of adding Bz to a dense cell culture (1×10^7^ cells/mL). After 2 h of cultivation, no significant decrease in CoQ_10_ level was observed following addition of Bz, and there was no significant change in the amount of CoQ_10_ (μg/50 mL medium) after treatment for 7 h (S5A Fig). From these observations, we concluded that addition of Bz did not promote the decomposition of CoQ.

In addition, we measured the amount of CoQ_10_ after long-term cultivation up to 75 h starting from 1.5×10^6^ cells/mL. Under these conditions, the amount of CoQ in cells reached the upper limit (~10.0 μg/10^9^ cells) without Bz, but it gradually increased following addition of Bz at 100 μg/mL (S5B Fig). This result indicates that although Bz inhibits CoQ biosynthesis, it does not completely block its synthesis.

Because the addition of Bz to YES complete medium lowered the pH to 5.6 from 6.0, we tested the effect of sodium benzoate (BzNa), which does not alter medium pH. The results revealed similar growth inhibition and decreases in the CoQ level for Bz and BzNa treatments at the same molar concentration (S6 Fig), suggesting that the decrease in pH caused by Bz treatment was not responsible for its negative effects on growth and the CoQ level in *S*. *pombe*.

### PABA or PHB can restore CoQ levels decreased by Bz

**Fig 4:**
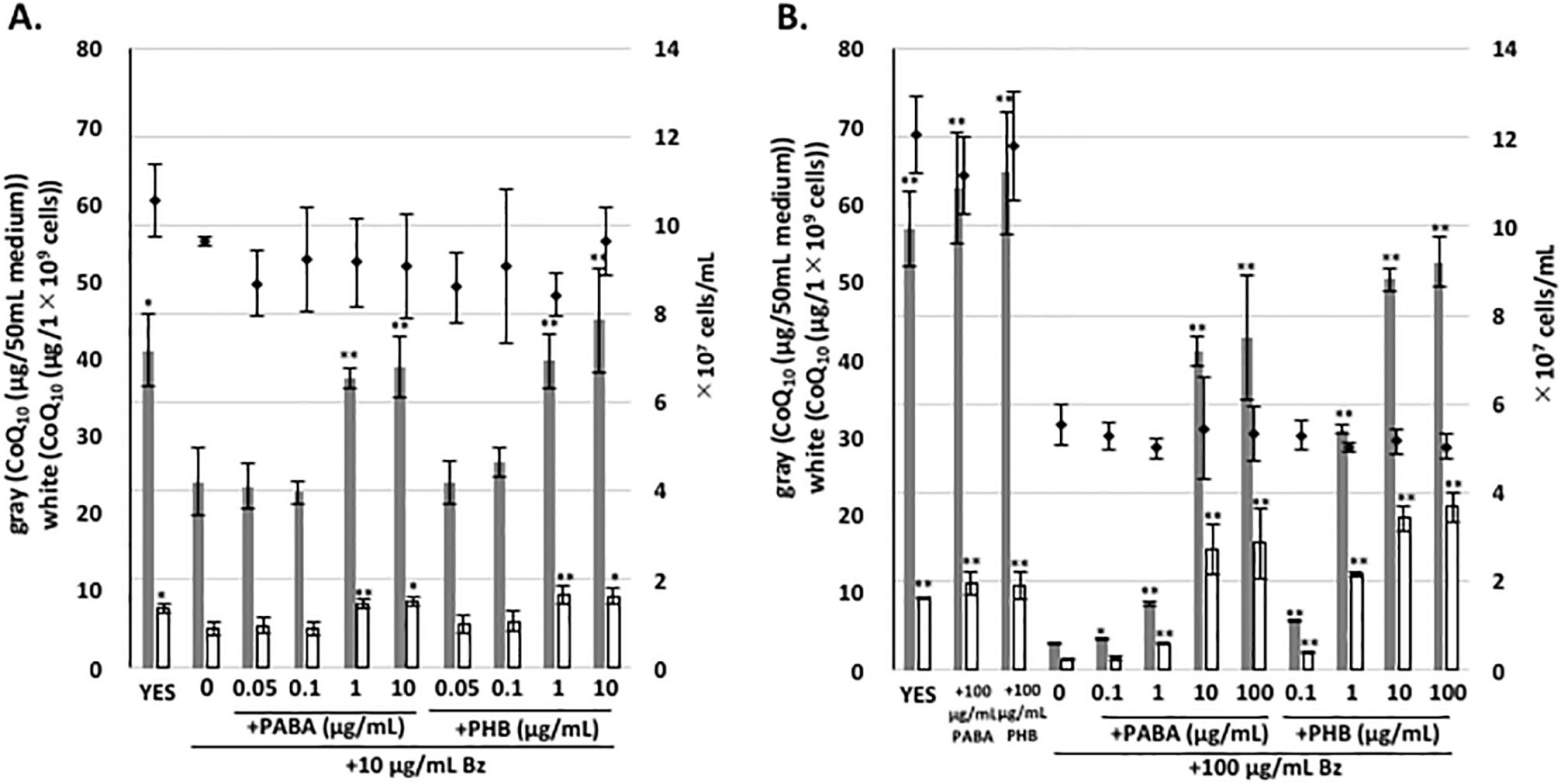
Reversible effect of PABA or PHB on inhibition of CoQ_10_ production by Bz. WT PR110 cells were pre-cultivated in 10 mL YES medium for 1 day. Cells at an initial density of ~1×10^5^ cells/mL were cultivated in the presence of 10 or 100 μg/mL Bz, or without Bz, as well as with PABA or PHB, with rotation at 30°C. Gray bars show the CoQ_10_ content per 50 mL of medium, and white bars show CoQ_10_ normalized against cell number. Diamonds show cell number. Five micrograms of CoQ_6_ was used as an internal standard. Data are represented as the mean ± SD of three measurements. Asterisks on bars denote statistically significant differences (**p<0.01, *p<0.05) relative to the sample in YES with 10 μg/mL Bz (A) or 100 μg/mL Bz (B), calculated by Student’s *t*-test.

We subsequently investigated the effect of PABA or PHB on the inhibition of CoQ_10_ biosynthesis by Bz. PABA or PHB (1 μg/mL) restored CoQ levels decreased by 10 μg/mL Bz treatment; 1 μg/mL (7.24 μM) PHB and 10 μg/mL (72.9 μM) PABA restored CoQ levels decreased by 100 μg/mL Bz treatment (Fig 4). This further indicates that PABA is utilized as a precursor in CoQ synthesis. PHB was more efficient than PABA at reversing the CoQ reduction following high-level Bz treatment. Co-treatment with PABA or PHB did not restore cell growth inhibited by Bz, indicating that Bz does not decrease CoQ levels by lowering cell growth. We did not observe any clear increase in CoQ_10_ production in *S*. *pombe* following treatment with PABA or PHB alone.

**Fig 5:**
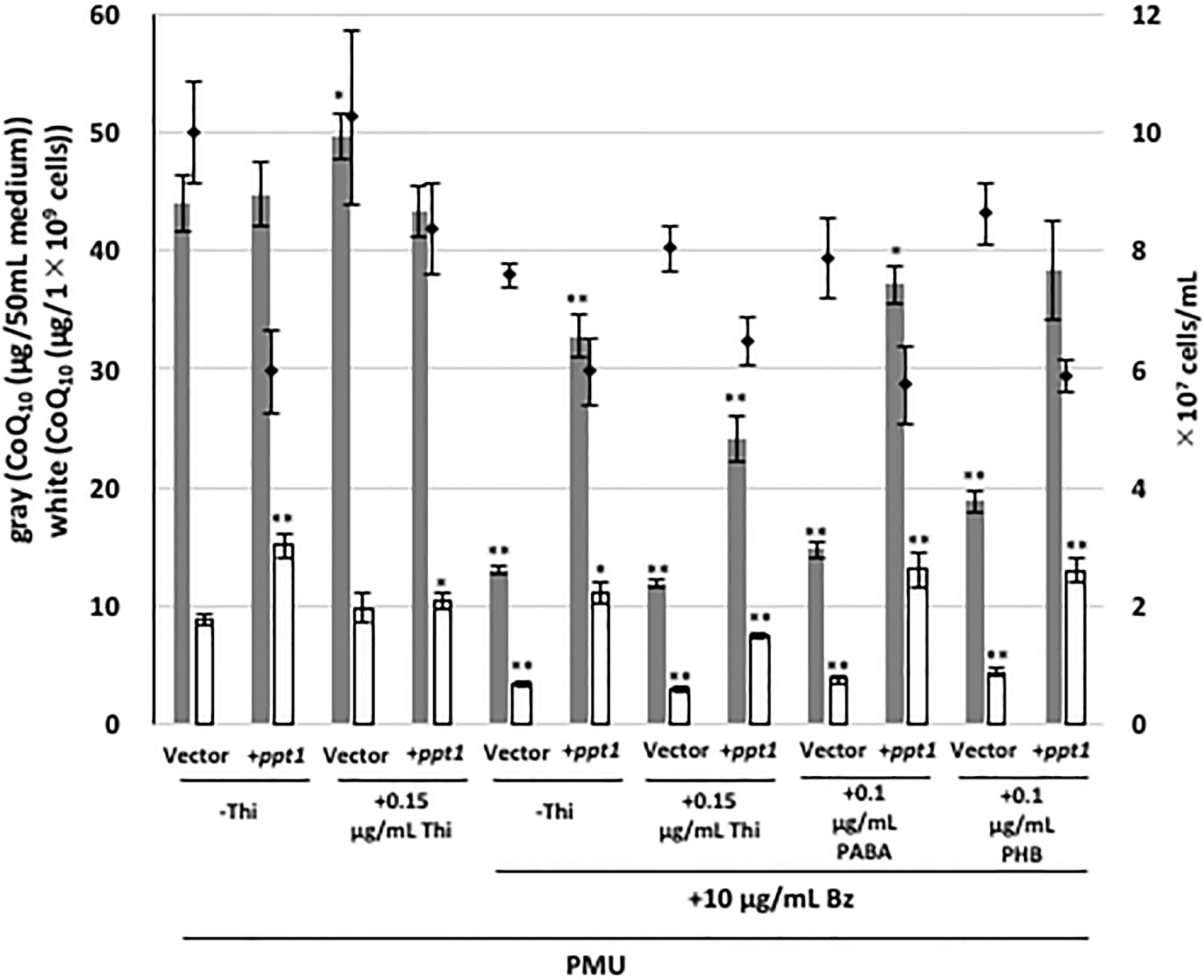
CoQ_10_ production by the *ppt1*-overexpressing strain treated with 10 μg/mL Bz. WT PR110 cells harboring pREP41 or pREP41-PPT1OR were cultivated in 10 mL PMU containing 0.15 μg/mL thiamine (Thi) for 1 day. Cells were washed three times with distilled water. Thiamine was added to repress the expression of the *nmt1* promoter, and 10 μg/mL of Bz and 0.1 μg/mL PABA/PHB were also added to the media containing ~1×10^6^ cells/mL and the cells were cultivated for the indicated time with rotation at 30°C. Gray bars show the CoQ_10_ content per 50 mL of medium, and white bars show CoQ_10_ normalized against cell number. Diamonds show cell number. Five micrograms of CoQ_6_ was used as an internal standard. Data are represented as the mean ± SD of three measurements. Asterisks on bars denote statistically significant differences (**p<0.01, *p<0.05) relative to the vector control sample without thiamine (Student’s *t*-test).

**Fig 6:**
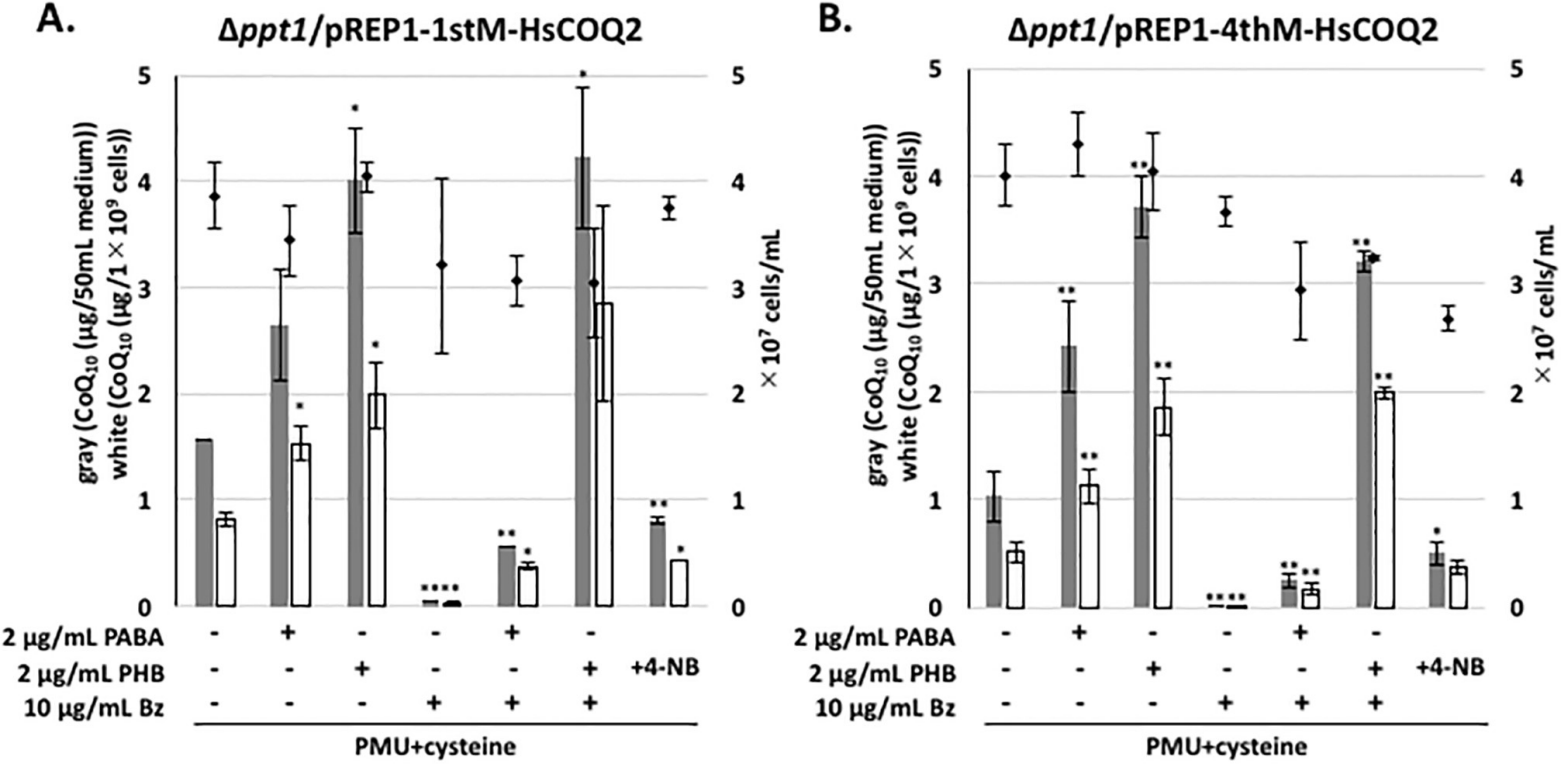
CoQ_10_ production by the human *COQ2*-expressing ∆*ppt1* strain after treatment with 10 μg/mL Bz or 4-NB. For the pre-culture, KH2 (Δ*ppt1*) yeast cells harboring pREP1-1stM-HsCOQ2 (A) or pREP1-4thM-HsCOQ2 (B) [10] were cultivated in 55 mL PMU medium containing 0.32 mg/mL cysteine and 0.15 μg/mL thiamine for 2 days. Cells were washed three times with distilled water and inoculated into 55 mL PMU medium containing 0.32 mg/mL cysteine (initial cell density ~1×10^6^ cells/mL) and cultivated for 2 days with rotation at 30°C. Next, 2 μg/mL PABA, 2 μg/mL PHB, 10 μg/mL Bz, or 100 μg/mL 4-NB was added to the media to test their effects. Gray bars show the CoQ_10_ content per 50 mL of medium, and white bars show CoQ_10_ normalized against cell number. Diamonds show cell number. Five micrograms of CoQ_6_ was used as an internal standard. Data are represented as the mean ± SD of two (A) or three (B) measurements. Asterisks on bars denote statistically significant differences (**p<0.01, *p<0.05) relative to PMU + cysteine (Student’s *t-*test).

It has been shown that analogs of PHB such as 4-NB can inhibit human Coq2 [22], suggesting that *S*. *pombe* Ppt1 (Coq2) might be a potential target of Bz. If this is the case, overexpression of *ppt1* (*coq2*) would counteract inhibition by Bz. To investigate CoQ production in the *ppt1* (*coq2*)-overexpressing strain, we employed plasmid pREP41-PPT1OR, which contains *ppt1* from *S*. *pombe* under the control of the *nmt1* thiamine-repressible promoter. As expected, *ppt1* overexpression abolished the decrease in the CoQ level caused by 10 μg/mL Bz treatment (Fig 5). Additionally, treatment with a lower concentration of PABA or PHB revealed an additive increase in CoQ production following *ppt1* overexpression in *S*. *pombe* in Bz-containing medium. In human, 4-NB inhibits CoQ biosynthesis, but the effect of Bz is unknown [22]. Therefore, a *ppt1* disruptant yeast strain expressing human *COQ2* (1stM-Hs*COQ2* and 4thM-Hs*COQ2* [10]) under the control of the *nmt1* thiamine-repressible promoter was used to test CoQ production following Bz or 4-NB treatment. When human *COQ2* was expressed in a fission yeast strain lacking *ppt1* (*coq2*), Bz inhibited CoQ production while 4-NB moderately inhibited CoQ production (Fig 6), and the addition of PABA or PHB restored CoQ production inhibited by Bz. This result indicates that Bz could potentially inhibit human CoQ production.

We next tested whether PABA is utilized in an artificial *S*. *pombe ppt1* (*coq2*) deletion strain expressing human *COQ2*. The results revealed that exogenously added 2 μM ^13^C_6_-PABA was effectively incorporated to produce CoQ_10_ in KH2 (Δ*ppt1*)/pREP1-1stM-HsCOQ2 and KH2 (Δ*ppt1*)/pREP1-4thM-HsCOQ2 strains, as well as in the WT strain (S7 Fig). Following addition of ^13^C_6_-PABA, CoQ_10_ levels in Δ*ppt1* strains expressing human *COQ2* were about four-fold higher than without PABA (S7 Fig). Utilization of PABA in human cells has not been confirmed, but our results indicate that human CoQ2 accepts PABA, and if the later pathway leading to CoQ is available, PABA would be utilized for CoQ synthesis in human.

### Phenotypic effects of Bz incubation

**Fig 7:**
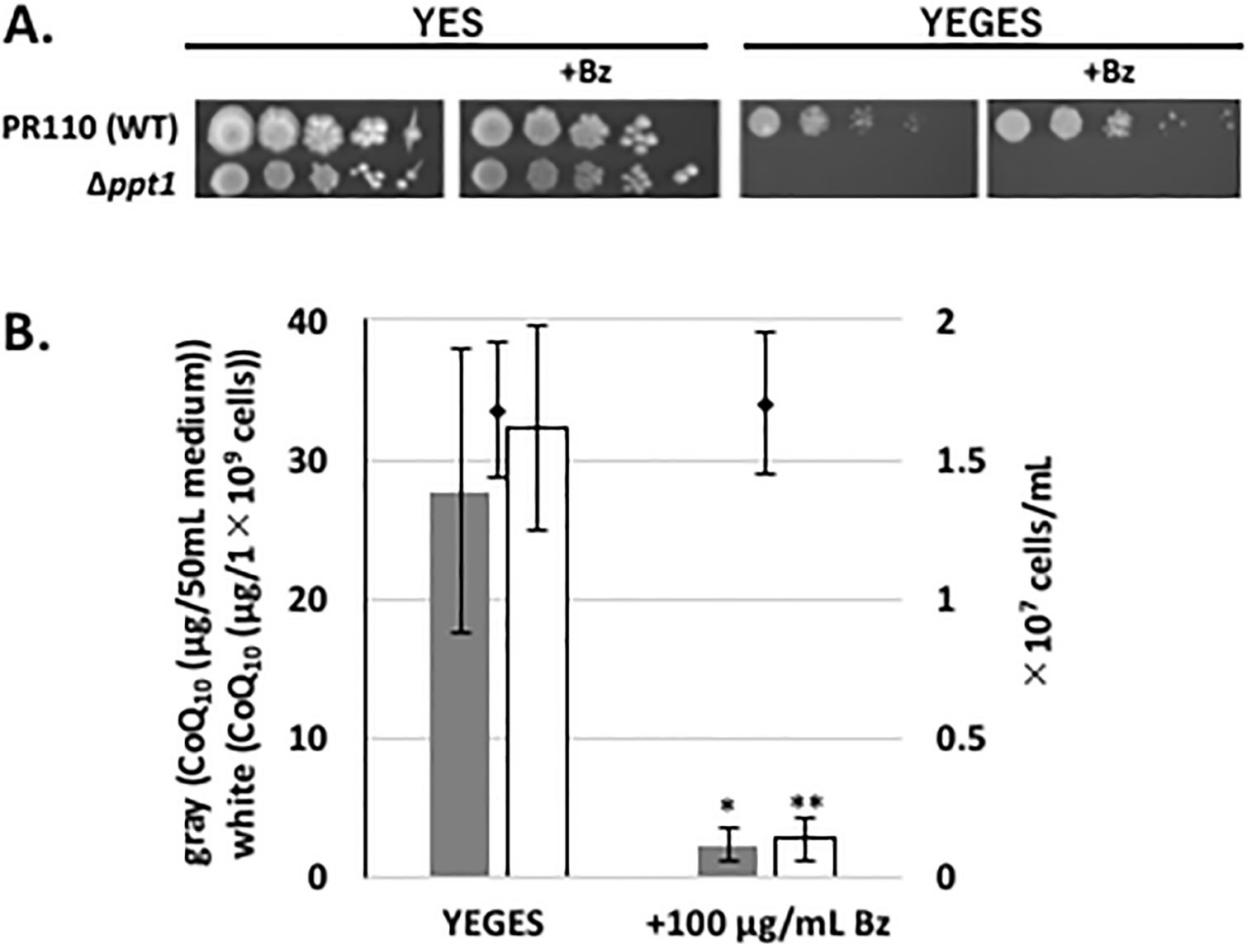
Growth and CoQ_10_ production of yeast growing on the non-fermentable carbon source YEGES following Bz treatment. (A) *S*. *pombe* strains were spotted onto YES or YEGES with or without 100 μg/mL Bz. Cells grown on YES for 1 day were washed three times. A culture with an OD_600_ of 2 was serially diluted from 10^−1^ to 10^−5^ (from left to right), spotted onto agar media, and cultured for 7 days. (B) For the pre-culture, PR110 yeast cells were cultivated in 55 mL of normal YES medium for 1 day, washed three times with pure water, and 100 μg/mL of benzoic acid (Bz) was added to YEGES media containing 2% (w/v) glycerol and 1% ethanol (w/v) instead of 3% glucose (w/v) with an initial cell density of ~1×10^7^ cells/mL, and cells were cultivated for 3 days at 30°C. Gray bars show the CoQ_10_ content per 50 mL medium, and white bars show CoQ_10_ normalized against cell number. Five micrograms of CoQ_6_ was used as an internal standard for CoQ extraction. Data are represented as the mean ± SD of three measurements. Asterisks on bars denote statistically significant differences (**p<0.01, *p<0.05) relative to YEGES without Bz (Student’s *t*-test).

CoQ-deficient mutants such as the *ppt1* (*coq2*) disruptant cannot grow on medium containing glycerol and ethanol as non-fermentable carbon sources [14, 38], but they can grow on medium containing a fermentative carbon source such as glucose (Fig 7A). We thought that Bz treatment may reduce growth on a medium containing a non-fermentable carbon source, due to reduction of CoQ synthesis. However, cell growth on non-fermentable media containing Bz was not distinguishable from that without Bz (Fig 7A). Also, the CoQ level was lower in cells grown in glycerol and ethanol with Bz than without (Fig 7B). Thus, Bz did not negatively affect cell growth in medium containing a non-fermentable carbon source in *S*. *pombe*.

CoQ is an electron acceptor for sulfide‐quinone oxidoreductase, and high production of sulfide is observed in CoQ-deficient fission yeast [39]. Therefore, the sulfide level under Bz treatment was tested, but it was not altered (S8 Fig). This is probably because inhibition by Bz does not completely abolish CoQ synthesis (Fig 3).

### Inhibition by Bz lowers Coq protein levels

**Fig 8:**
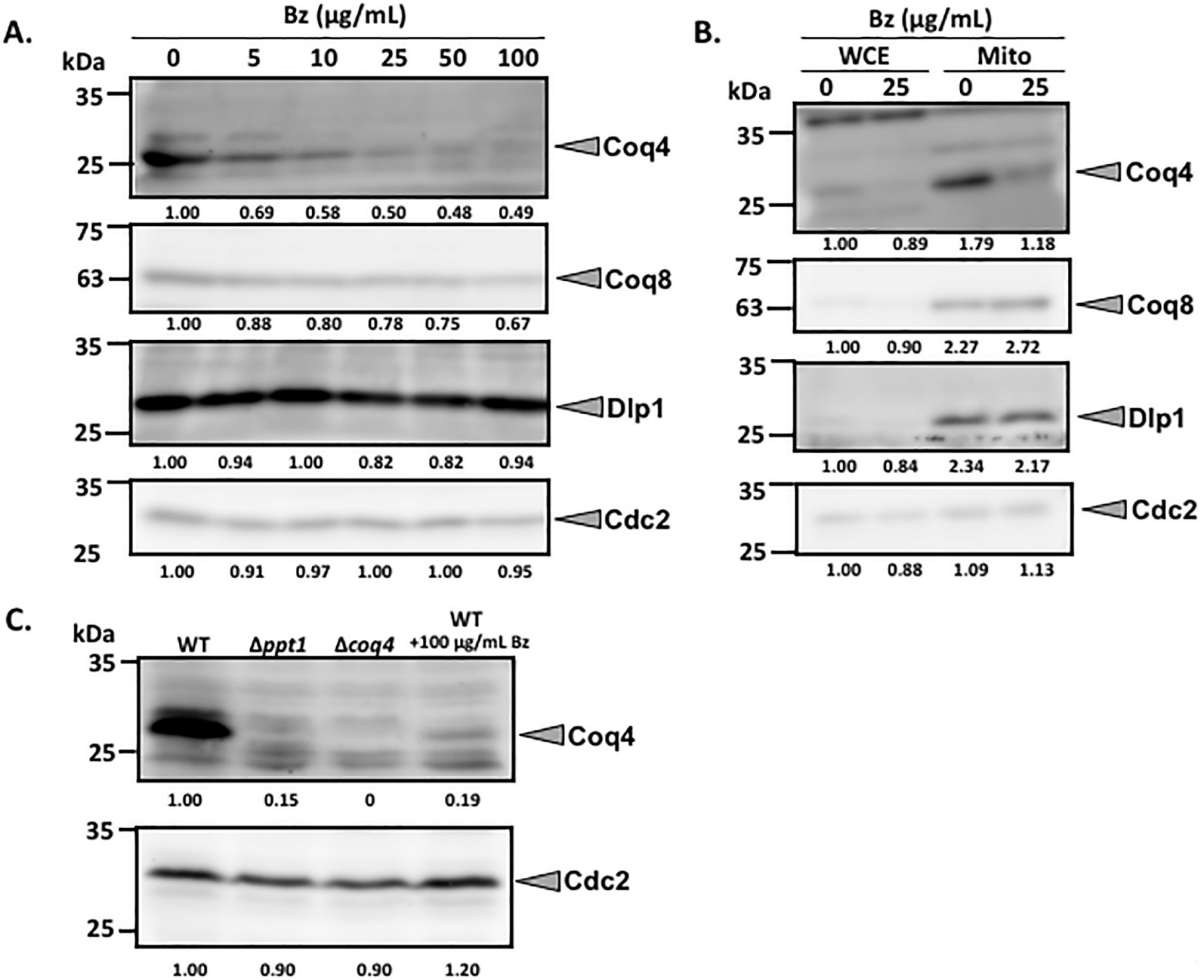
Western blotting of Coq4, Coq8, and Dlp1 under various Bz concentrations. (A) Coq4, Coq8, Dlp1, and Cdc2 as a loading control for whole cells were analyzed by western blotting. Target proteins are indicated on the right. The concentrations of Bz in each lane are shown at the top. For the pre-culture, PR110 yeast cells were cultivated in 10 mL YES for 1 day. Yeast cells were cultivated in 55 mL YES at an initial cell density of 1×10^5^ cells/mL and cultivated for 48 h with rotation at 30°C. (B) Mitochondria were isolated as described in Materials and Methods. A 5 μg protein sample from the whole cell extract (WCE) or purified mitochondria (Mito)-enriched samples were used (right panel). (C) WT (PR110), Δ*ppt1* (KH2), and Δ*coq4* (KH4) strain with 100 μg/mL of Bz were cultivated in the YES media containing ~1×10^5^ cells/mL for two days with rotation at 30°C. Then, Coq4 and Cdc2 proteins were detected by western blotting as described above. The amount of proteins was quantified by Image J.

It has been shown that the biosynthetic enzymes responsible for CoQ form a multi-enzyme complex in *S*. *cerevisiae* [40], and Coq4 is the central organizer [41, 42]. We believe that the same may be true for *S*. *pombe*, based on our preliminary data. Therefore, the effect of Bz on Coq protein levels was analyzed, and the results showed that the Coq4 and Coq8 proteins decreased after adding ≥5 μg/mL Bz (Fig 8A). However, the Dlp1 protein level was not changed by Bz treatment. A similar trend of low abundance of the Coq4 protein, but not the Coq8 protein, by Bz inhibition was observed in isolated mitochondria (Fig 8B). When the abundance of the Coq4 protein was tested in Δ*ppt1* strain, it was a comparable level of wild type cells incubated with 100μg/mL Bz (Fig 8C), which support the idea that Bz inhibits the Ppt1 (Coq2) reaction. Overexpression of the *coq4* gene did not restore the production of CoQ_10_ reduced by Bz inhibition (S9 Fig), therefore, it is unlikely that the reduction of the Coq4 protein is a sole reason for lowering CoQ_10_ production by Bz. We think that a decrease in Coq protein expression destabilizes the Coq multi-enzyme complex, but further studies employing antibodies specific for other Coq proteins will be needed to test this hypothesis.

### Inhibition of CoQ synthesis in other microorganisms

**Fig 9:**
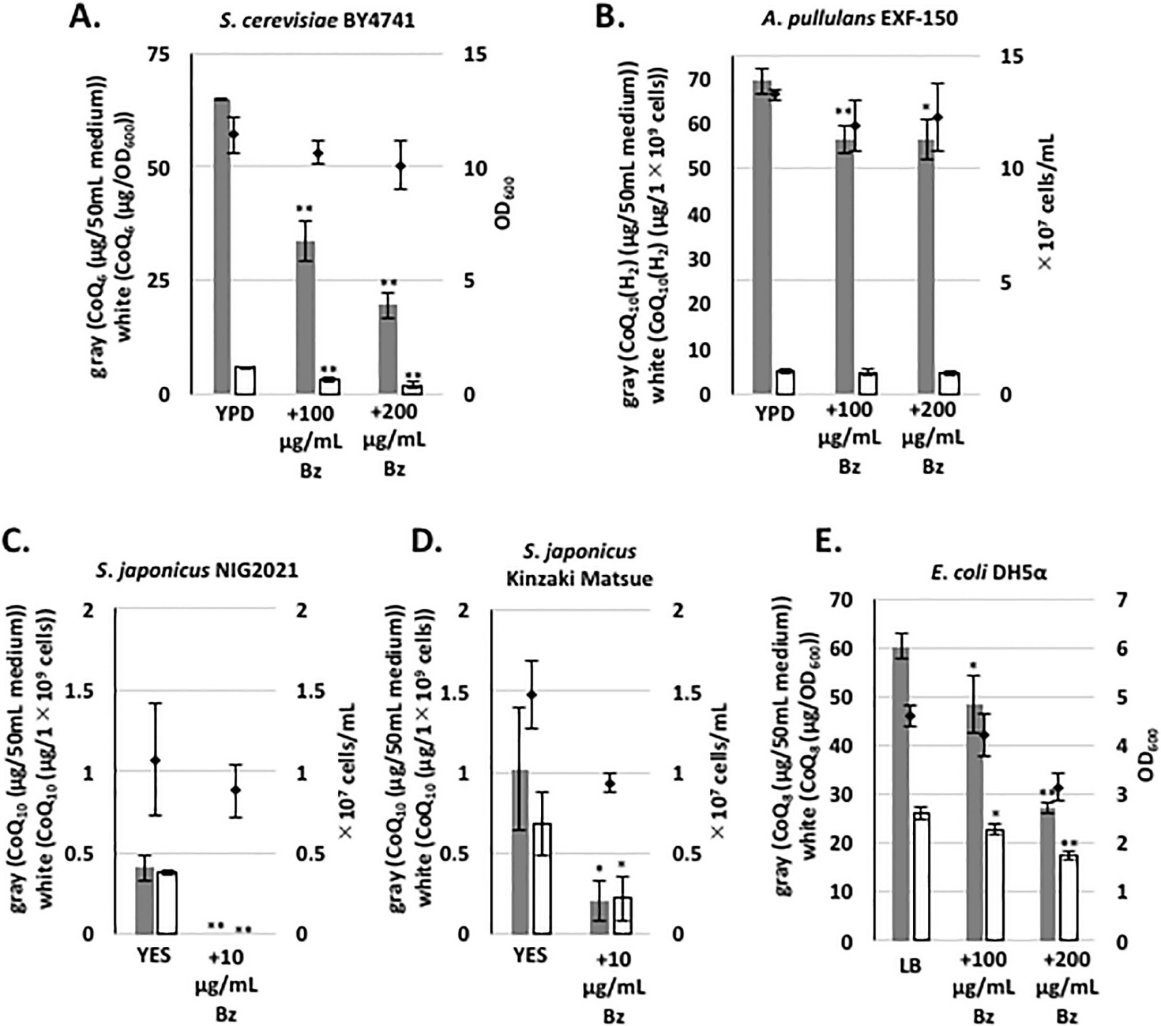
CoQ production in various microorganisms following Bz addition. For the pre-culture, *S*. *cerevisiae* BY4741, *A*. *pullulans* EXF-150 [43], *S*. *japonicus* NIG2021, *S*. *japonicus* isolated from a Kinzaki ancient tomb located in Matsue [38], and *E*. *coli* DH5α cells were cultivated in 10 mL of the indicated medium for 1 day. To explore the inhibitory effect of Bz, the indicated amount (μg/mL) of Bz was added to the media. For fungi, the initial cell density was ~1×10^5^ cells/mL and cells were cultivated for 2 days with rotation at 30°C; for *E*. *coli*, the initial cell density was OD_600_ 0.1 and cells were cultivated for 12 h with rotation at 37°C. Gray bars show the CoQ_10_ content per 50 mL of medium, and white bars show CoQ_10_ normalized against cell number. Diamonds show cell number or optical density. Five micrograms of CoQ_6_ was used as an internal standard for measuring CoQ_8_, CoQ_10_, or CoQ_10_(H_2_), which is CoQ_10_ with a saturated isoprenoid unit in the side chain. Five micrograms of CoQ_10_ was used as an internal standard for measuring CoQ_6_. Data are represented as the mean ± SD of three measurements. Asterisks on bars denote statistically significant differences (**p<0.01, *p<0.05) relative to each medium without Bz (Student’s *t*-test).

We next explored whether Bz inhibits CoQ synthesis in other microorganisms. The effect of Bz was moderate in *S*. *cerevisiae*, even at a concentration of 100 μg/mL (Fig 9A), and no inhibition was observed in *A*. *pullulans* (Fig 9B). Inhibition of CoQ synthesis by Bz was clearly observed at a 10-fold lower concentration (10 μg/mL) in *S*. *japonicus* using two independent strains (Fig 9C and 9D), although the amount of CoQ was very low in these species. The effect of Bz on *E*. *coli* was also moderate (Fig 9E). Thus, inhibition by Bz is much more efficient in *S*. *pombe* and *S*. *japonicus* than in *S*. *cerevisiae* and *E*. *coli*.

## Discussion

In the present study, we showed that PABA is utilized for CoQ synthesis in *S*. *pombe*, as was demonstrated previously for *S*. *cerevisiae*. PHB is commonly utilized as a precursor of CoQ in both prokaryotes and eukaryotes [8]. However, exactly how widely PABA is utilized for CoQ synthesis is not yet clear. For example, human and *E*. *coli* do not utilize PABA for CoQ synthesis, probably because the pathway to modify the prenylated PABA leading to the synthesis of CoQ is lacking [24]. It has been reported that exogenous PABA is prenlyated by prenlytransferase in mammalian tissues [24], hence mammalian COQ2 must be able to conjugate PABA with polyprenyl diphosphate. When we examined the effect of PABA in the *S*. *pombe ppt1* (*coq2*) deletion strain expressing human *COQ2*, PABA counteracted the inhibitory effect of Bz on the synthesis of CoQ. Since *S*. *pombe* possesses the pathway to synthesize CoQ from PABA, human *COQ2* appears to be able to prenylate PABA. Furthermore, replacing *S*. *pombe ppt1* (*coq2*) with human *COQ2* made it possible to synthesize CoQ from PABA. Although utilization of PABA as a precursor of CoQ in human cells has not been proved, our results indicate that human COQ2 accepts PABA as a substrate.

This is the first study to report the effect of Bz on CoQ synthesis in *S*. *pombe*. We think that Bz is likely to be an inhibitor of prenylation of PABA and PHB by Ppt1 (Coq2) for two reasons. Firstly, inhibition by Bz was reversed by an ~10-fold lower concentration of PABA and PHB, and this inhibition was overcome by overexpression of *ppt1* (*coq2*) gene. These observations support the idea that Bz targets Ppt1 (Coq2). In previous reports, *in vitro* assay analysis of the prenylation of several compounds indicated that PABA, vanillic acid, and protocatechuic acid are prenlylated in rat [24]. Although Bz was not tested in this experiment, Coq2 has a broad substrate spectrum and accepts a wide range of related compounds.

Addition of Bz lowered the abundance of the Coq4 protein. This suggests that once the enzymatic reaction of CoQ synthesis is halted by an inhibitor, at least the Coq4 protein becomes unstable (Fig 8). We did not see such an effect in the Dlp1 protein, presumably because Dlp1 is separated from the complex of CoQ synthesis in *S*. *pombe*. *S*. *pombe* likely forms a complex of CoQ synthetic enzymes (our unpublished observations). The enzymatic complex responsible for CoQ synthesis, named the Q synthome, has been well studied in *S*. *cerevisiae* [18], and PHB stabilizes the Q synthome [42]. It has also been shown that the expressions of *COQ* genes including *COQ4* in *S*. *cerevisiae* is not affected by loss of Q synthome formation [44]. All together suggest the proper formation of the CoQ synthetic enzyme complex affects the protein stability of Coq4, but not the expression of *coq4*, in *S*. *pombe*. Further studies are needed to reveal more about complex stability.

Bz clearly inhibits CoQ synthesis in *S*. *japonicus*, although the amount of CoQ is very low in this species (~100 times lower than in *S*. *pombe*) [38]. We observed moderate inhibition of CoQ by Bz in *S*. *cerevisiae* and *E*. *coli*, but almost no inhibition in *A*. *pullulans*. We think that differences in inhibition are not due to the specificity of Coq2, because we observed inhibition by Bz in *S*. *pombe* cells expressing human *COQ2*. COQ2 and its homolog are interchangeable among species; *S*. *cerevisiae COQ2* is functionally exchangeable with UbiA in *E*. *coli* [45] and an *Arabidopsis PPT1* (*COQ2*) homolog with *S*. *cerevisiae COQ2* [46]. If the specificity of Bz to various Coq2 homologs is not so strict, differences in the inhibitory effect of Bz on CoQ synthesis among different organisms might be due to differences in how effectively Bz is transported inside cells [47] and into mitochondria. On the contrary, the observation in this study that an inhibitory effect of 4-NB was not observed in *S*. *pombe* but observed in *S*. *pombe* having replaced with human *COQ2*, might suggest this difference is due to the difference in substrate recognition among Coq2 homologs. To clarify these aspects, further studies will be required for precise inhibitory mechanism of these compounds.

Bz and BzNa have been widely used as food additives to inhibit the growth of microorganisms in foods and soft drinks [48, 49]. Bz is considered generally safe at a concentration up to 0.1%, which is 10 times higher than 100 μg/mL concentration employed in this study. At a concentration of 100 μg/mL of Bz, growth of *S*. *pombe* was clearly inhibited, but not that of *S*. *cerevisiae* and *A*. *pullulans*. We again speculate that differences in growth inhibition among the tested species may be due to differences in the uptake efficiency of this compound, resulting in differences in the inhibitory effect of Bz on CoQ synthesis. While plants synthesize Bz [50, 51], yeasts do not, and how Bz is metabolized in yeasts is not well understood. In yeasts, at least in *S*. *pombe*, Bz is an unfavorable compound for cell growth.

In conclusion, we demonstrated that PABA is efficiently utilized as a precursor of CoQ synthesis in *S*. *pombe*. Bz inhibits *S*. *pombe* CoQ synthesis, presumably by inhibiting the PHB/PABA prenyl transferase enzyme encoded by *ppt1* (*coq2*).

## Supporting information

S1 Fig CoQ biosynthesis in *S*. *pombe*. In this study, PABA was shown to be utilized as a precursor for a quinone ring in addition to PHB in *S*. *pombe*. Decaprenyl diphosphate, which is synthesized by decaprenyl diphosphate synthase (Dps1 + Dlp1), is transferred to PABA or PHB by PABA/PHB-decaprenyl diphosphate transferase (Ppt1, Coq2), and the aromatic ring is then modified during CoQ biosynthesis. DAB, 5-decaprenyl-4-aminobenzoic acid; DHB, 5-decaprenyl-4-hydroxybenzoic acid; DPP, decapentenyl diphosphate; FPP, farnesyl diphosphate; IPP, isopentenyl diphosphate; PABA, *p*-aminobenzoic acid; PHB, *p*-hydroxybenzoic acid.
(TIFF)

S2 Fig Chemical structures of benzoic acid and its related compounds used in this study. (TIFF)

S3 Fig CoQ_10_ production by L972 yeast cells following Bz treatment. For the pre-culture, WT L972 yeast cells were cultivated in 10 mL medium for 1 day. Cells (initial cell density 1×10^5^ cells/mL) were grown with or without 100 μg/mL of Bz and cultivated for 2 days with rotation at 30°C. Gray bars show the CoQ_10_ content per 50 mL medium, and white bars show CoQ_10_ normalized against cell number. Five micrograms of CoQ_6_ was used as an internal standard for CoQ extraction. Data are represented as the mean ± SD of three measurements. Asterisks on bars denote statistically significant differences (**p<0.01) relative to YES without Bz.
(TIFF)

S4 Fig Colony Forming Unit (CFU) and CoQ_10_ production in the WT strain treated with 10 μg/mL and 100 μg/mL Bz. (A) The PR110 strain was pre-cultivated in 10 mL YES for 1 day. Cells were grown with 10 μg/mL or 100 μg/mL of Bz in 70 mL new media containing ~1×10^5^ cells/mL, and cultivated for two days with rotation at 30°C. Cell number was measured by Sysmex cell counter and diluted 10^4^ times. 100 μL of each sample was plated onto YES plates and CFU was counted after incubation for 3–4 days. (B) CoQ_10_ production of the cells used in (A). Gray bars show the CoQ_10_ content per 50 mL of medium, and white bars show CoQ_10_ normalized by cell number. Diamonds show cell number. Five micrograms of CoQ_6_ was used as an internal standard. Data are represented as the mean ± SD of three measurements. Asterisks on bars denote statistically significant differences (**p<0.01) relative to YES.
(TIFF)

S5 Fig CoQ_10_ production at various timepoints following Bz treatment. For the pre-culture, PR110 yeast cells were cultivated in 55 mL medium for 1 day. Cells at an initial cell density of 1×10^7^ cells/mL (A) or 1.5×10^6^ cells/mL (B) were grown with or without 100 μg/mL of benzoic acid (Bz) and cultivated for the indicated time with rotation at 30°C. Gray bars show the CoQ_10_ content per 50 mL medium, and white bars show CoQ_10_ normalized against cell number. Five micrograms of CoQ_6_ was used as an internal standard for CoQ extraction. Data are represented as the mean ± SD of three measurements. Asterisks on bars denote statistically significant differences (**p<0.01) relative to the 0 h (A) or 12 h timepoint (B) without Bz (Student’s *t*-test).
(TIFF)

S6 Fig CoQ_10_ production in the presence of various concentrations of Bz or BzNa. For the pre-culture, WT PR110 cells were cultivated in 10 mL medium for 1 day. The indicated amount (μg/mL) of Bz or sodium benzoate (BzNa) was added to the media (initial cell density ~1×10^5^ cells/mL) and cultivated for the indicated time with rotation at 30°C. Gray bars show the CoQ_10_ content per 50 mL of medium, and white bars show CoQ_10_ normalized against cell number. Diamonds show cell number. Five micrograms of CoQ_6_ was used as an internal standard. Data are represented as the mean ± SD of three measurements.
(TIFF)

S7 Fig Utilization of PABA in CoQ_10_ synthesis in the ∆*ppt1* strain expressing human *COQ2*. For the pre-culture, WT PR110 yeast cells harboring pREP1, KH2 (Δ*ppt1*) harboring pREP1-1stM-HsCOQ2, or pREP1-4thM-HsCOQ2 were cultivated in 10 mL PMU medium containing 0.32 mg/mL cysteine and 0.15 μg/mL thiamine for 2 days. Cells were washed three times with distilled water and inoculated into 55 mL PMU medium containing 0.32 mg/mL cysteine (initial cell density ~2×10^6^ cells/mL) and cultivated for 1 day with rotation at 30°C. A 2 μg/mL sample of ^13^C_6_-PABA was added to confirm the incorporation to the quinone ring of CoQ. CoQ_10_-enriched samples were obtained after separation of lipids by TLC, and samples were subjected to LC-MS and LC-MS/MS (daughter scan) analyses to detect stable isotope-labeled CoQ_10_. In PR110/pREP1 (A), KH2 (Δ*ppt1*)/ pREP1-1stM-HsCOQ2 (B), and KH2 (Δ*ppt1*)/pREP1-4thM-HsCOQ2 (C) strains, samples were prepared with and without 2 μg/mL ^13^C_6_-PABA and analyzed by LC-MS/MS). The amount of CoQ_10_ is shown in (D). Gray bars show the CoQ_10_ content per 50 mL of medium, and white bars show CoQ_10_ normalized against cell number. Diamonds show cell number. Five micrograms of CoQ_6_ was used as an internal standard.
(TIF)

S8 Fig H_2_S concentration in WT, WT grown in 100 μg/mL Bz, and Δ*ppt1* strain. Yeast cells were grown in YES for indicated times and H_2_S concentrations were measured by the method described previously [39].
(TIFF)

S9 Fig CoQ_10_ production by the *coq4*-overexpressing strain treated with 10 μg/mL or 100 μg/mL Bz. WT PR110 cells harboring pREP1 (Vector) or pREP1-Spcoq4 (+*coq4*) [31] were cultivated in 10 mL PMU containing 0.15 μg/mL thiamine for 1 day. 0.15 μg/mL thiamine was added to repress the expression of the *nmt1* promoter, and 10 μg/mL and 100 μg/mL of Bz were also added to the media containing ~1×10^6^ cells/mL and the cells were cultivated for one day with rotation at 30°C. Gray bars show the CoQ_10_ content per 50 mL of medium, and white bars show CoQ_10_ normalized against cell number. Diamonds show cell number. Five micrograms of CoQ_6_ was used as an internal standard. Data are represented as the mean ± SD of two measurements.
(TIFF)

S1 Table LC-MS conditions. (DOCX)

S1 Raw image (ZIP)
