# Supplementary figures and images for: Benzoic acid inhibits Coenzyme Q biosynthesis in Schizosaccharomyces pombe
Source: PLoS One. 2020 Nov 24;15(11):e0242616. doi: 10.1371/journal.pone.0242616 (PMC7685456; doi:10.1371/journal.pone.0242616)

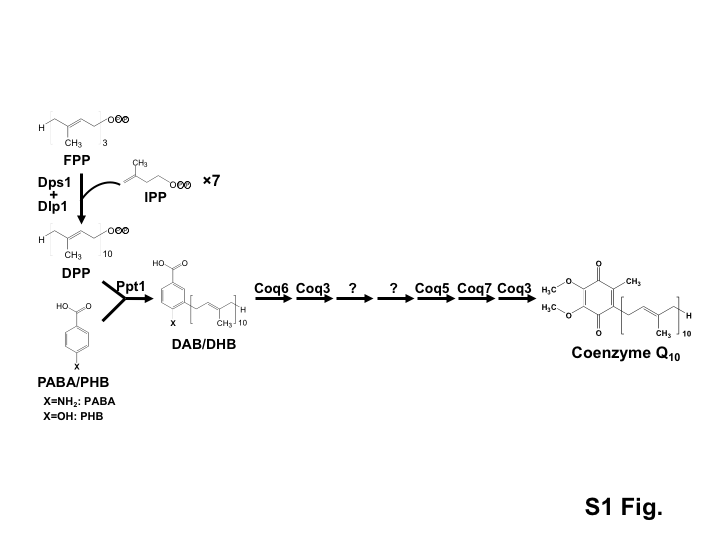

Supplement: S1 Fig — In this study, PABA was shown to be utilized as a precursor for a quinone ring in addition to PHB in S. pombe. Decaprenyl diphosphate, which is synthesized by decaprenyl diphosphate synthase (Dps1 + Dlp1), is transferred to PABA or PHB by PABA/PHB-decaprenyl diphosphate transferase (Ppt1, Coq2), and the aromatic ring is then modified during CoQ biosynthesis. DAB, 5-decaprenyl-4-aminobenzoic acid; DHB, 5-decaprenyl-4-hydroxybenzoic acid; DPP, decapentenyl diphosphate; FPP, farnesyl diphosphate; IPP, isopentenyl diphosphate; PABA, p-aminobenzoic acid; PHB, p-hydroxybenzoic acid. (TIFF) [file pone.0242616.s001.tiff]

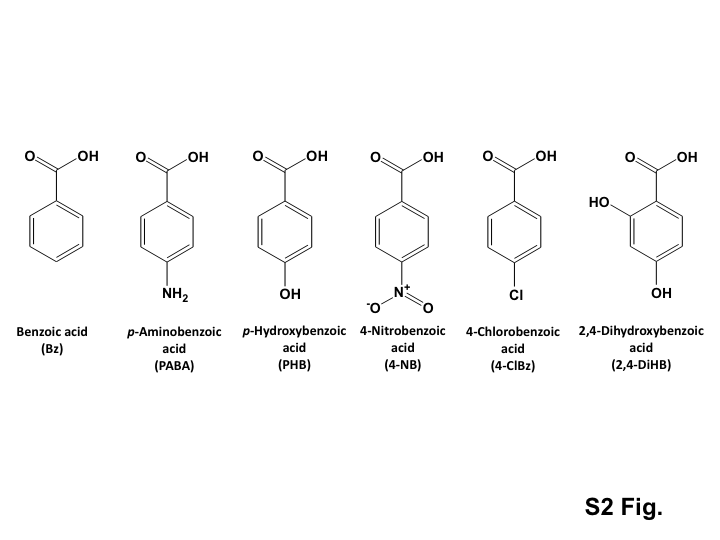

Supplement: S2 Fig — (TIFF) [file pone.0242616.s002.tiff]

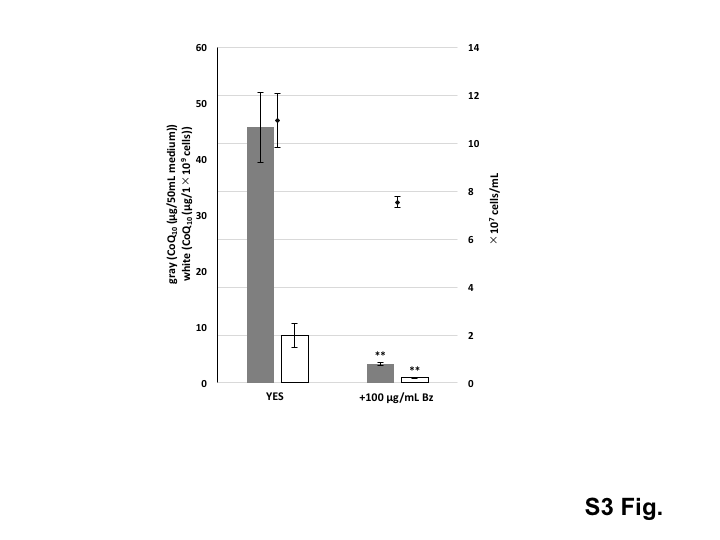

Supplement: S3 Fig — For the pre-culture, WT L972 yeast cells were cultivated in 10 mL medium for 1 day. Cells (initial cell density 1×10^5^ cells/mL) were grown with or without 100 μg/mL of Bz and cultivated for 2 days with rotation at 30°C. Gray bars show the CoQ10 content per 50 mL medium, and white bars show CoQ10 normalized against cell number. Five micrograms of CoQ6 was used as an internal standard for CoQ extraction. Data are represented as the mean ± SD of three measurements. Asterisks on bars denote statistically significant differences (**p<0.01) relative to YES without Bz. (TIFF) [file pone.0242616.s003.tiff]

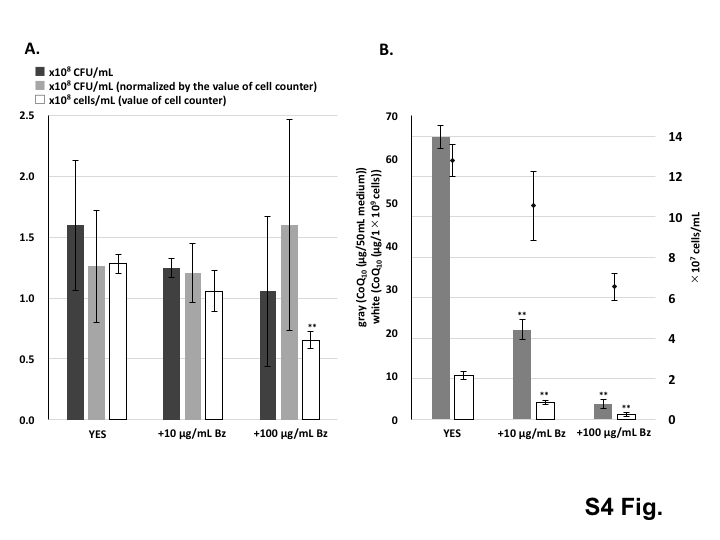

Supplement: S4 Fig — (A) The PR110 strain was pre-cultivated in 10 mL YES for 1 day. Cells were grown with 10 μg/mL or 100 μg/mL of Bz in 70 mL new media containing ~1×10^5^ cells/mL, and cultivated for two days with rotation at 30°C. Cell number was measured by Sysmex cell counter and diluted 104 times. 100 μL of each sample was plated onto YES plates and CFU was counted after incubation for 3–4 days. (B) CoQ10 production of the cells used in (A). Gray bars show the CoQ10 content per 50 mL of medium, and white bars show CoQ10 normalized by cell number. Diamonds show cell number. Five micrograms of CoQ6 was used as an internal standard. Data are represented as the mean ± SD of three measurements. Asterisks on bars denote statistically significant differences (**p<0.01) relative to YES. (TIFF) [file pone.0242616.s004.tiff]

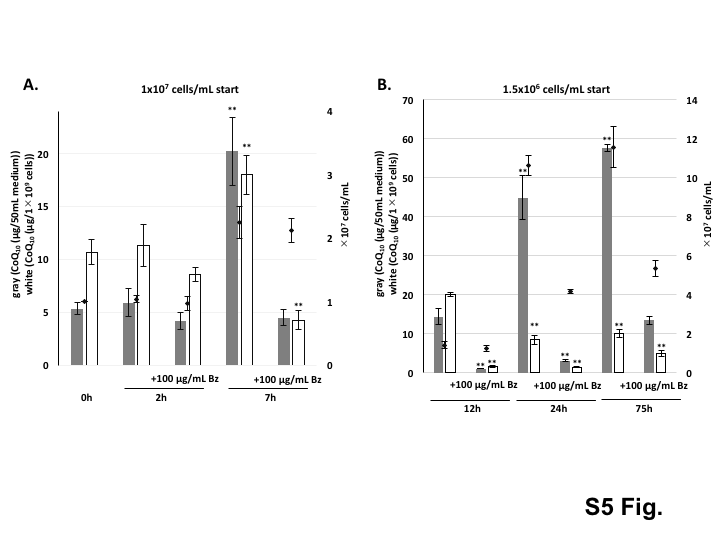

Supplement: S5 Fig — For the pre-culture, PR110 yeast cells were cultivated in 55 mL medium for 1 day. Cells at an initial cell density of 1×10^7^ cells/mL (A) or 1.5×10^6^ cells/mL (B) were grown with or without 100 μg/mL of benzoic acid (Bz) and cultivated for the indicated time with rotation at 30°C. Gray bars show the CoQ10 content per 50 mL medium, and white bars show CoQ10 normalized against cell number. Five micrograms of CoQ6 was used as an internal standard for CoQ extraction. Data are represented as the mean ± SD of three measurements. Asterisks on bars denote statistically significant differences (**p<0.01) relative to the 0 h (A) or 12 h timepoint (B) without Bz (Student’s t-test). (TIFF) [file pone.0242616.s005.tiff]

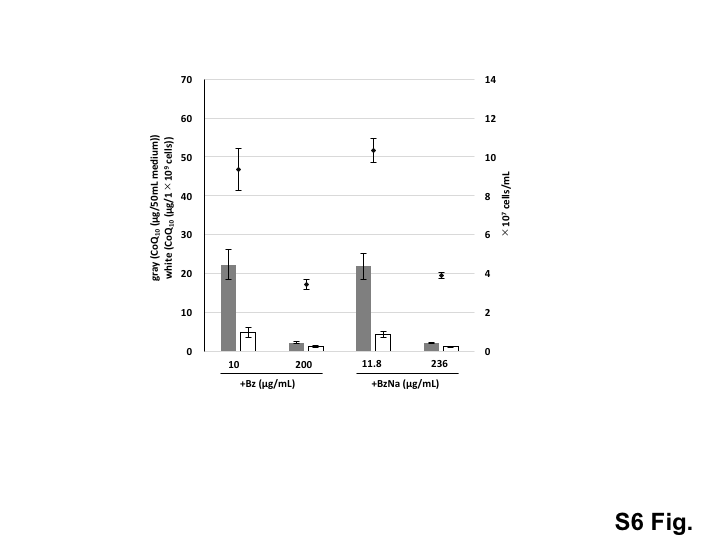

Supplement: S6 Fig — For the pre-culture, WT PR110 cells were cultivated in 10 mL medium for 1 day. The indicated amount (μg/mL) of Bz or sodium benzoate (BzNa) was added to the media (initial cell density ~1×10^5^ cells/mL) and cultivated for the indicated time with rotation at 30°C. Gray bars show the CoQ10 content per 50 mL of medium, and white bars show CoQ10 normalized against cell number. Diamonds show cell number. Five micrograms of CoQ6 was used as an internal standard. Data are represented as the mean ± SD of three measurements. (TIFF) [file pone.0242616.s006.tiff]

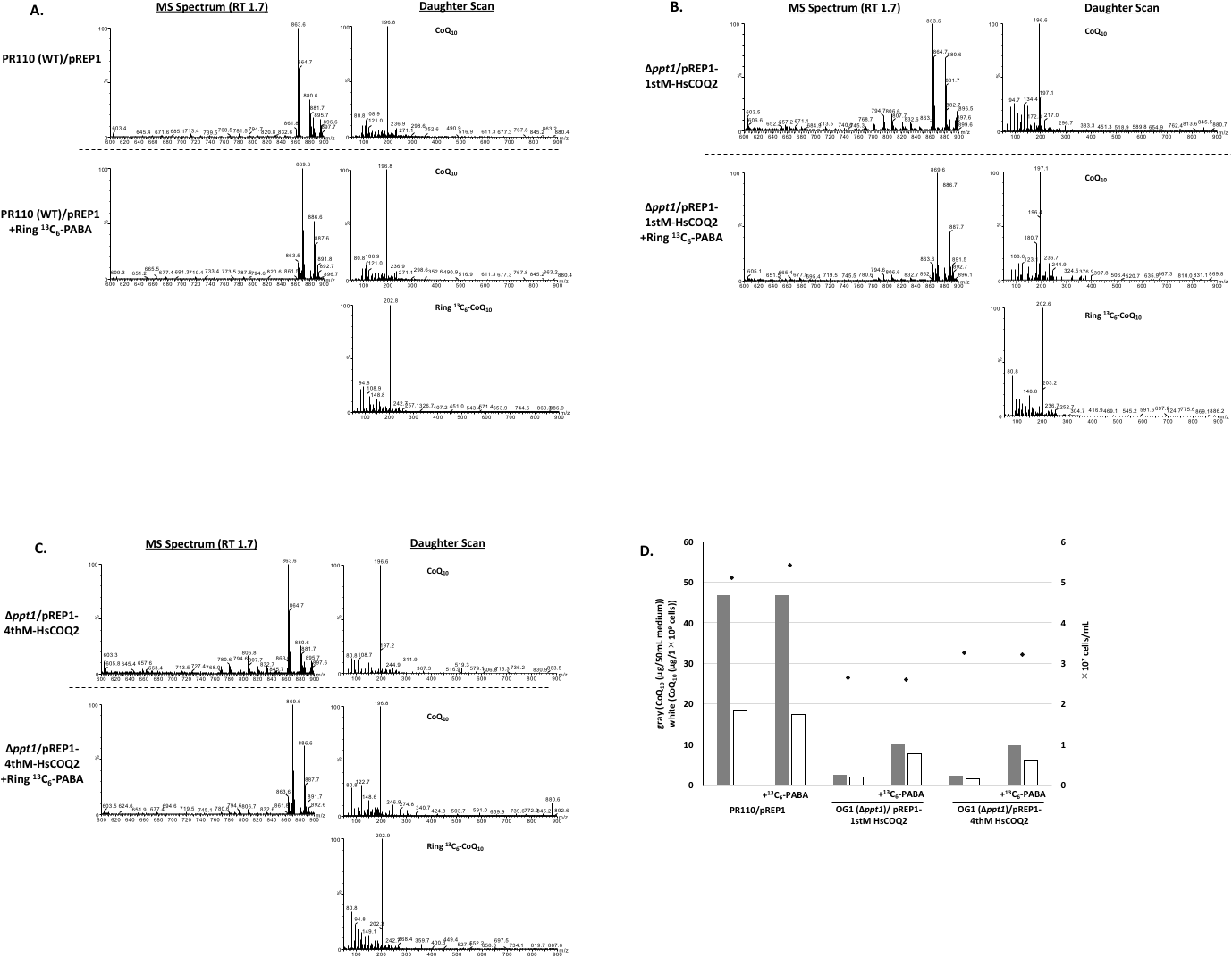

Supplement: S7 Fig — For the pre-culture, WT PR110 yeast cells harboring pREP1, KH2 (Δppt1) harboring pREP1-1stM-HsCOQ2, or pREP1-4thM-HsCOQ2 were cultivated in 10 mL PMU medium containing 0.32 mg/mL cysteine and 0.15 μg/mL thiamine for 2 days. Cells were washed three times with distilled water and inoculated into 55 mL PMU medium containing 0.32 mg/mL cysteine (initial cell density ~2×10^6^ cells/mL) and cultivated for 1 day with rotation at 30°C. A 2 μg/mL sample of 13C6-PABA was added to confirm the incorporation to the quinone ring of CoQ. CoQ10-enriched samples were obtained after separation of lipids by TLC, and samples were subjected to LC-MS and LC-MS/MS (daughter scan) analyses to detect stable isotope-labeled CoQ10. In PR110/pREP1 (A), KH2 (Δppt1)/ pREP1-1stM-HsCOQ2 (B), and KH2 (Δppt1)/pREP1-4thM-HsCOQ2 (C) strains, samples were prepared with and without 2 μg/mL 13C6-PABA and analyzed by LC-MS/MS). The amount of CoQ10 is shown in (D). Gray bars show the CoQ10 content per 50 mL of medium, and white bars show CoQ10 normalized against cell number. Diamonds show cell number. Five micrograms of CoQ6 was used as an internal standard. (TIF) [file pone.0242616.s007.tif]

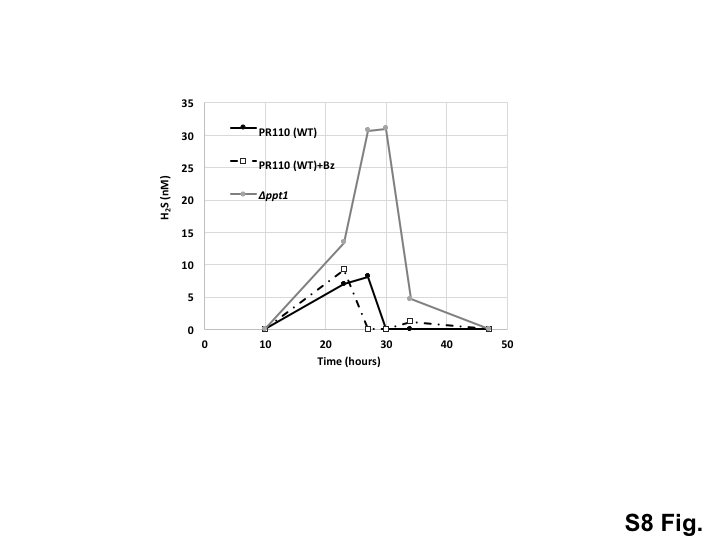

Supplement: S8 Fig — Yeast cells were grown in YES for indicated times and H2S concentrations were measured by the method described previously [39]. (TIFF) [file pone.0242616.s008.tiff]

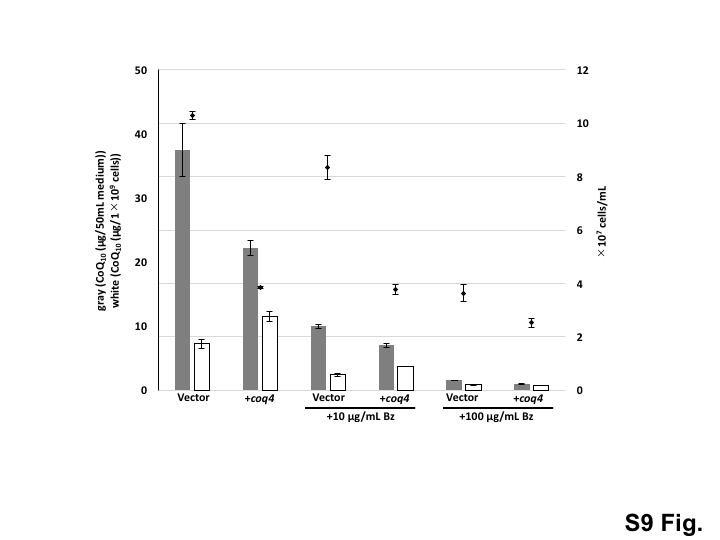

Supplement: S9 Fig — WT PR110 cells harboring pREP1 (Vector) or pREP1-Spcoq4 (+coq4) [31] were cultivated in 10 mL PMU containing 0.15 μg/mL thiamine for 1 day. 0.15 μg/mL thiamine was added to repress the expression of the nmt1 promoter, and 10 μg/mL and 100 μg/mL of Bz were also added to the media containing ~1×10^6^ cells/mL and the cells were cultivated for one day with rotation at 30°C. Gray bars show the CoQ10 content per 50 mL of medium, and white bars show CoQ10 normalized against cell number. Diamonds show cell number. Five micrograms of CoQ6 was used as an internal standard. Data are represented as the mean ± SD of two measurements. (TIFF) [file pone.0242616.s009.tiff]
