# Supplementary material for: Benzoic acid inhibits Coenzyme Q biosynthesis in Schizosaccharomyces pombe
Source: PLoS One. 2020 Nov 24;15(11):e0242616. doi: 10.1371/journal.pone.0242616 (PMC7685456; doi:10.1371/journal.pone.0242616)
Supplement: S1 Table — (DOCX) [file pone.0242616.s010.docx]

**S1 Table. LC-MS conditions.**

----------------------------------------------------------------------------------------------------

HPLC system Acquity UPLC System (Waters)

Column ACQUITY UPLC BEH C18, 130Å, 1.7 µm

(2.1 mm x 50 mm column, Part No. 186002350)

Mobile phase Buffer A and B (80% methanol v/v for LC/MS, 20%

2-propanol v/v for LC/MS, containing100 μM ammonium

formate) where eluent A is 50%, and eluent B is 50%

Flow rate 0.5 mL/min

UV 190−400 nm

Column temperature 30°C

Sample temperature 10°C

Injection volume 8 μL

----------------------------------------------------------------------------------------------------

MS/MS system Xevo TQ MS (Waters)

Ionization mode APCI-Positive (ESCi^+^)

Calibration Dynamic 2

Corona 30.00 μA, 3.5 kV

Capillary 2.98 kV

Cone 30 V

Source temperature 130°C

Desolvation temperature 400°C

Cone gas flow OFF

Desolvation gas Nitrogen, 800 L/h

Collision gas flow 0.15

Product ion scan of *m*/*z* 600−900 (MS), 100-900 (MS/MS)

Scan speed 1 s

Collision energy 40 eV (MS/MS)

-----------------------------------------------------------
